# Towards an inclusive and culturally sensitive conceptualisation of sexual well-being of young people: preliminary framework development using a modified Delphi methodology

**DOI:** 10.1080/26410397.2025.2474337

**Published:** 2025-03-05

**Authors:** Lore Remmerie, Guncha Annageldiyeva, Kayleigh Grossman, Caesar Kaba Kogoziga, Nicole Leonetti, Ana Mosiashvili, Shreya Shrestha, Tisungane Sitima, Evi Stuckens, Michael Tetteh Doku, Aslan Temirkhanov, Diana Marcela Zambrano, Heidi Mertes, Kristien Michielsen

**Affiliations:** a PhD Researcher, Institute for Family and Sexuality Studies, Department of Neurosciences, Faculty of Medicine, KU Leuven, Leuven, Belgium.; b Y-PEER International Coordinator, Y-PEER International Network, Ashgabat, Turkmenistan; c Masters by Research Candidate, School of Anthropology and Conservation, University of Kent, Canterbury, UK; d Programmes Coordinator, Planned Parenthood Association of Ghana, Accra, Ghana; e Youth Advocate, CHOICE for Youth and Sexuality, Amsterdam, The Netherlands; f Secretary-General, European Confederation of Youth Clubs, Brussels, Belgium; g Student, Erasmus+ Scholar Women and Gender Studies (GEMMA); University of lodz/ Utrecht University, Kathmandu, Nepal; h Youth worker, Foundation for Rural Development, Blantyre, Malawi; i Volunteer, Sensoa VZW, Antwerp, Belgium; j Advocacy and Communications Manager, Curious Minds Ghana, Accra, Ghana; k Independent Researcher, Nazarbayev University School of Medicine, Astana, Kazakhstan; l Independent Researcher, Research Department, Profamilia, Bogotá, Colombia; m Associate Professor, Department of Philosophy and Moral Sciences and Department of Public Health and Primary Care, Ghent University, Ghent, Belgium; n Associate Professor, Institute for Family and Sexuality Studies, Department of Neurosciences, Faculty of Medicine, KU Leuven, Leuven, Belgium

**Keywords:** sexual well-being, positive approach, SRHR, participatory research, youth, Delphi method

## Abstract

This study aims to contribute to the development of a comprehensive framework for sexual well-being of young people. By making space for diverse young people’s perspectives through co-creating the framework, we seek to enhance the understanding of sexual well-being in sexual and reproductive health and rights (SRHR) research in a culturally sensitive and inclusive way. A modified Delphi study invited SRHR young professionals (aged 18–30) with different backgrounds to participate as experts in three rounds of online discussions. A framework of sexual well-being was co-created by 15 young professionals from countries across Europe, Asia, Africa, and South America. This framework recognised sexual well-being as a subjective concept with different meanings: for example, by applying an open understanding of sexual activity, and intimacy. It also acknowledged the challenges individuals face in fully understanding and achieving their sexual well-being, due to societal injustices. The framework outlined key capabilities inherent to sexual well-being, including informed decision-making, bodily autonomy, consent, exploration, self-awareness, pleasure, communication, comfort, safety, and self-esteem. Considering that these capabilities can only be realised within an enabling environment, access to sexual health information and services, as well as acceptance, respect, safety, and freedom from coercion and violence, were included as a key part of the framework. This study captured young people’s views on sexual well-being to co-create a culturally sensitive framework. This framework recognises different interpretations of sexual well-being, and focuses on supportive environments that empower individuals to define and pursue sexual well-being in a way that honours their experiences and needs.

## Plain language summary

Discussions on what sexual well-being means and how to measure it are important for setting future priorities. The discussions are mainly happening among academics, leaving out important groups like young people, local communities, and the LGBTQIA+ community. These groups deserve to be part of the conversation, so we created a platform for their views to be shared. We worked with 15 young professionals from around the world to design a sexual well-being framework. We held three rounds of discussions: using a shared online board, an online meeting, and a feedback session. This gave the young experts time to think and rethink their ideas. A sexual well-being framework for young people was designed jointly. The framework recognises that sexual well-being is different for everyone and that we should not judge different understandings. We should continue learning what sexual well-being means to different people. We also acknowledge that society can make it hard for young people to define and achieve sexual well-being. Sexual well-being has a more universal component which includes being in a supportive environment where young people can make informed choices, control their body, explore, experience pleasure, communicate, feel safe, understand their sexual health, and build self-esteem. This research is a first step towards giving voice to youth perspectives in defining sexual well-being. We hope this motivates researchers to include more voices, making sexual and reproductive health research more inclusive and culturally sensitive.

## Résumé

Cette étude a pour but de contribuer à l’élaboration d’un cadre global pour le bien-être sexuel des jeunes. En faisant de la place aux perspectives diverses des jeunes, grâce à la création collective du cadre, nous cherchons à faciliter la compréhension du bien-être sexuel dans la recherche sur la santé et les droits sexuels et reproductifs d’une manière inclusive et culturellement sensible. Une étude Delphi modifiée a invité de jeunes professionnels de la santé et des droits sexuels et reproductifs (âgés de 18 à 30 ans) venant d’horizons différents à participer en tant qu’experts à trois séances de discussions en ligne. Un cadre de bien-être sexuel a été cocréé par 15 jeunes professionnels de pays d’Europe, d’Asie, d’Afrique et d’Amérique du Sud. Ce cadre reconnaît le bien-être sexuel comme un concept subjectif avec différentes significations, par exemple en appliquant une compréhension ouverte de l’activité sexuelle et de l’intimité. Il prend aussi en compte les difficultés que les individus rencontrent pour comprendre et réaliser pleinement leur bien-être sexuel, en raison des injustices sociétales. Le cadre met en exergue les principales capacités inhérentes au bien-être sexuel, notamment la prise de décision éclairée, l’autonomie corporelle, le consentement, l’exploration, la conscience de soi, le plaisir, la communication, le confort, la sécurité et l’estime de soi. Étant donné que ces capacités ne peuvent être réalisées que dans un environnement propice, l’accès aux informations et aux services de santé sexuelle, ainsi que l’acceptation, le respect, la sécurité et la protection contre la coercition et la violence ont été inclus comme éléments clés du cadre. Cette étude a recueilli les points de vue des jeunes sur le bien-être sexuel pour cocréer un cadre culturellement sensible. Ce cadre prend en considération différentes interprétations du bien-être sexuel et se concentre sur des environnements favorables qui donnent aux individus les moyens de définir et de rechercher leur bien-être sexuel d’une manière qui honore leurs expériences et leurs besoins.

## Resumen

Este estudio pretende contribuir a la creación de un marco integral para el bienestar sexual de las personas jóvenes. Al hacer espacio para las diversas perspectivas de jóvenes por medio de la cocreación del marco, buscamos mejorar la comprensión del bienestar sexual en las investigaciones sobre salud y derechos sexuales y reproductivos (SDSR) de una manera culturalmente sensible e inclusiva. Un estudio Delphi modificado invitó a jóvenes profesionales en SDSR (de 18 a 30 años) con diferentes antecedentes a participar como expertos en tres rondas de debates en línea. Un marco de bienestar sexual fue cocreado por 15 jóvenes profesionales provenientes de países en Europa, Asia, África y Sudamérica. Este marco reconoció el bienestar sexual como un concepto subjetivo con diferentes significados, por ejemplo, al aplicar una comprensión abierta de la actividad e intimidad sexual. También reconoció los retos que enfrenta cada persona para entender y lograr plenamente su bienestar sexual, debido a las injusticias sociales. El marco resumió las capacidades clave inherentes al bienestar sexual, tales como una toma de decisiones informada, autonomía corporal, consentimiento, exploración, autoconciencia, placer, comunicación, comodidad, seguridad y autoestima. Considerando que estas capacidades solo pueden realizarse dentro de un entorno facilitador, el acceso a información y servicios de salud sexual, así como aceptación, respeto, seguridad y libertad de coerción y violencia, fueron incluidos como una parte fundamental del marco. Este estudio capturó los puntos de vista de jóvenes sobre el bienestar sexual para cocrear un marco culturalmente sensible, que reconoce las diferentes interpretaciones del bienestar sexual y se enfoca en entornos de apoyo que empoderan a cada persona para definir y perseguir el bienestar sexual de una manera que honra sus experiencias y necesidades.

## Introduction

The World Health Organization (WHO) recognises that sexual health is crucial for individuals, families, communities, and countries.^1^ Although great progress has been made over the past decades, integrating sexual and reproductive health and rights (SRHR) into the global political agenda has been a challenging process.^2^ Reaching consensus around a common SRHR language has been an important part of the global debates.^3^ The understanding of sexual health has evolved over the past decades, with the most recent WHO working definition of sexual health dating from 2006.^4^

*“Sexual health is a state of physical, emotional, mental and social well-being in relation to sexuality; it is not merely the absence of disease, dysfunction or infirmity. Sexual health requires a positive and respectful approach to sexuality and sexual relationships, as well as the possibility of having pleasurable and safe sexual experiences, free of coercion, discrimination and violence. For sexual health to be attained and maintained, the sexual rights of all persons must be respected, protected and fulfilled.”* ^5^

Compared to its earlier versions, the definition considers well-being in relation to sexuality and emphasises the importance of a holistic, positive and comprehensive approach, rather than the traditional medical approach focusing on the absence of disease. However, researchers have been slow to embrace this broader perspective, often still concentrating on sexual dysfunction and reproduction, and less on well-being.^6,7^ By linking sexual rights to sexual health, the definition also recognises the importance of applying a rights-based approach to sexual health. This means recognising the critical role of fundamental human rights, such as non-discrimination, equality, and rights to freedom of opinion, in achieving sexual health.^1^ The definition states that “the sexual rights of all persons must be respected, protected, and fulfilled”, highlighting the need for inclusive research practices that capture the unique SRHR needs of all groups. However, key populations – such as young people, local communities, and LGBTQIA+ individuals – remain underrepresented in research.^8^ While some progress has been made, these groups still require greater attention to close knowledge gaps and resolve persisting inequities. This underscores the need for continued debate to refine SRHR priorities and language, and to promote more positive and rights-based approaches in SRHR practices.

Numerous academics have advocated to integrate a “positive approach” into SRHR research, programmes, and service delivery.^9–12^ The Guttmacher–Lancet Commission states that a positive approach involves acknowledging the importance of pleasurable sexual relationships, trust, and communication for self-esteem and overall well-being, alongside the fundamental right to autonomy over one’s own body.^8^ It means honouring diverse sexual behaviours, gender identities and sexual orientations, and challenging traditional notions of what is considered “normal” or “healthy”.^13–16^ Especially in the field of young people’s SRHR, where research and education programmes have largely focused on the negative consequences of sex, experts emphasise the need to recognise these positive aspects as equally important for young people’s healthy sexual development.^11^ The concept of sexual well-being has been introduced to enhance the positive approach.^14,15^

The concept gained more recognition after it was discussed in 2007 by a WHO working group, with the aim to offer a broader interpretation of sexual health. Considering the cultural and context-specific nature of sexual well-being, the WHO refrained from providing a definition or specifying measures for this concept. Instead, they underscored the necessity to conduct further research to enhance the understanding of sexual well-being and formulate appropriate measures.^17^ The lack of conceptual clarity does not align with needs in the academic field where the interest in the concept of sexual well-being is clearly growing. A review of 162 papers on the concept of sexual well-being identified major gaps in its conceptualisation.^18^ Definitions of sexual well-being were frequently lacking, and measurements were often limited to sexual function. Further, most authors used individual assessments, focusing less on sociocultural aspects such as gender norms and cultural norms around sexuality, thereby failing to capture the complexity of the concept. Finally, little attention is given to contextual differences in this concept.^18,19^ Yet again, similarly to the concept of sexual health, it can be concluded that the way sexual well-being is currently conceptualised impedes expansion to a broader, rights-based, and more positive approach to sexual health research.

An inclusive framework for measuring sexual well-being could help to stimulate a positive and rights-based approach to SRHR research and programmes, but how to implement this is still up for debate. Recognising the potential benefits of sexual well-being in the public health field, some researchers have provided a starting point for the operationalisation of the concept of sexual well-being.^4,15,20^

Mitchell et al. consider sexual well-being as a crucial indicator of both population well-being and health equity.^20^ They introduced a seven-domain model to conceptualise and operationalise sexual well-being, distinguishing it from sexual health. Building on this model, the seven-item scale (Natsal-SW) was developed to assess sexual well-being in the UK context.^21^ Lorimer et al. emphasised the importance of integrating capabilities into the concept of sexual well-being to be able to consider the power dynamics, politics, and patriarchy that shape sexual and reproductive health and rights.^19^ Building on this, Kågesten and van Reeuwijk created a competency-based framework designed for adolescent sexual development.^15^ This framework identified key competencies that help adolescents navigate their environments and achieve sexual well-being. De Meyer et al. identified the aspect of social (sexual) well-being, highlighting the role societal norms play in shaping adolescents’ sexual experiences.^22^

These frameworks provide an interesting foundation for advancing the concept of sexual well-being. However, as noted by Lorimer et al., it is important to ensure the concept of sexual well-being is rooted in lived realities of different groups of people.^19^ This is also confirmed by Kågesten and van Reeuwijk, who noted that most data and existing frameworks come from high-income Western countries.^15^ They recommend refining and validating the framework by engaging with key stakeholders, including young people, in different settings globally.

By emphasising the value of including diverse views, the authors make an important point. As mentioned before, different perspectives, including those of young people, have been under-acknowledged in SRHR research.^7,8^ Therefore, it seems relevant, and fundamental to a rights-based approach, to include specifically these voices to guide decision-making and priority-setting. This is especially important when developing a framework for sexual well-being, which intends to consider the context-specificity and cultural sensitivity of the concept. Young people, with their unique positionality and life experiences, might shine a unique light on the conceptualisation of sexual well-being. Their voices have often been overlooked in decision-making processes, especially in SRHR.^23^ The United Nations acknowledges the role of young people as driving forces of change and wants to empower youth-led organisations to engage in translating the 2030 Sustainable Development Goal agenda.^24^ We believe that engaging diverse groups of young people in the conceptualisation of sexual well-being, can empower them to take on their role as active agents of change.

This research aims to guide future research and programmes by developing a preliminary framework that conceptualises sexual well-being of young people in a culturally sensitive and inclusive way, thereby promoting positive and rights-based SRHR practices. By actively involving a diverse group of young individuals, we enable youth voices to have a meaningful impact on the direction of SRHR work. Additionally, this study investigates strategies for researchers to better incorporate diverse youth perspectives when developing frameworks in a global context.

## Materials & methods

### Modified Delphi design

We used an adapted Delphi methodology, a method designed to bring experts together to discuss a certain topic in multiple rounds with the goal of reaching consensus.^25,26^ The reason for using a multiple-round Delphi design, rather than a qualitative study at one time point, was to allow participants to gain a deeper understanding of the topic over time and allow them to refine their opinions. Delphi designs are currently used in multiple disciplines and can be adapted and modified for several research aims.^27^ Constructivist principles guided our decisions regarding the modifications made to the Delphi design. This paradigm assumes the existence of multiple realities and emphasises the importance of understanding individuals and how they interpret the surrounding world.^28^ We find alignment between this paradigm and our research objectives, as we believe that defining sexual well-being in a culturally sensitive way involves striving to understand and respect its diverse interpretations. Other authors have also emphasised the alignment of constructivist principles with the aims of Delphi methods, particularly in health and well-being research.^29^ Guzys et al. argue that Delphi methods cannot meet the traditionally applied positivistic criteria, which aim for objectivity and generalisability. Constructivist principles, which acknowledge the subjectivity of expert opinions, are therefore considered more appropriate.^29^

The following modification were made:

The Delphi method typically involves a quantitative aspect, applying statistical aggregation to compile group responses.^27^ As the objective was not to establish a rigid consensus on sexual well-being, such as a generalisable understanding accompanied by an exhaustive set of metrics for measurement, but rather to give meaning to differences in opinions, including the opinions of minorities, and think about ways to incorporate these differences into a conceptual framework, it was decided to only use qualitative methods for data collection.

The term “Experts” in a Delphi study is traditionally seen as individuals offering different perspectives due to their extensive professional expertise. We argue that this overlooks the subjectivity inherent in researchers’ opinions. Standpoint theory asserts that expert opinions are shaped by personal, social, cultural, and historical contexts.^30^ Intersectionality theory recognises individuals’ multiple intertwined social positions, contributing to complex expertise drawn from unique life experiences.^31^ While often considered professionally as “less experienced”, young adults offer valuable insights from direct involvement in sexual and reproductive health matters. Hence, this research takes an unconventional stance on “expertise”, recruiting individuals not only for their professional engagement but also for their diverse and often underrepresented personal experiences in SRHR.

### Participants and recruitment

There is no agreement on what sample size is adequate for a panel in a Delphi study, but good results can already be achieved in panels from 10 to 15 individuals.^32^ When panels are too large, this can have a negative impact on profound participation of all members in all parts of the research process.

Participants, aged 18–30 years, were recruited between October and December 2022. Although the framework was designed to conceptualise sexual well-being of young people, we decided to include the older age range (18–24 years old) and slightly older participants to gain valuable reflections from those able to look back on their youth. Young individuals with some knowledge and understanding of SRHR through education, activism, research, or participation in sexual health oriented-organisations were targeted, as we assumed that these groups might feel more comfortable openly communicating about a sensitive topic like sexual well-being. This way we aimed to ensure that perspectives from societies where discussions about sexuality are less common would not be overshadowed or overridden by viewpoints from societies where discussions about sexuality are more openly embraced. Recruitment focused on diversity, targeting organisations from different countries and working with minority groups, including gender and sexual minorities as well as people with disabilities. Although efforts were made to include a range of minority groups, the selection cannot fully represent all demographics. Proficiency in English was required. Recruitment involved reaching out to 43 SRHR organisations (Annexe 1) via email to disseminate study details. Interested individuals attended an online introductory meeting with LR, where detailed information was shared, questions answered, and input on the research topic and methodology was welcomed. These meetings aimed to connect, enhance participation, assess English proficiency, and ensure informed consent. Seventeen individuals expressed interest through email, 15 attended introductory meetings, and signed consent forms, and began the study. These participants will now be referred to as “panel members”.

### Data collection

Data were collected in three rounds between December 2022 and April 2023. A progress report was shared with the panel members to stay up to date with the project management, to provide an outline for the different rounds, and to give the panel members the chance to share reflections and ideas throughout the process. A detailed overview of the questions in the different rounds can be found in Annexe 2. All rounds were facilitated by the first author.

#### Round 1

In the initial round, panel members engaged in an online asynchronous brainstorming exercise on Padlet to explore their understanding of sexual well-being. This method allowed for deliberate reflections and community discussion. Anonymous responses were made visible for everyone to facilitate discussion.

#### Round 2

During the second round, three two-hour online focus group discussions took place. The discussions were guided by semi-structured, open-ended questions, based on results from the brainstorm and previous studies.^18,33,34^ Panel members were asked to reflect on three example definitions/descriptions of sexual well-being.^17,35,36^ We selected these definitions based on Lorimer et al.’s ideas^18^ because we believe they effectively illustrate the key variations among different conceptualisations of sexual well-being: one focusing more on subjective well-being, one on capabilities,^36^ and one on a combination.^17^ Visual slides on the interactive Miro platform served as the guiding tool during the discussions. A summary of the discussions was shared with all panel members, including the ones that did not participate online, with the option to add additional thoughts to the discussions. Some panel members were contacted for clarifications through email. These emails were also included as data.

#### Round 3

Following the second-round analysis, a draft conceptual framework and definition of sexual well-being were presented in a written report. This included qualitative analysis, quotes, interpretations, and a visual framework. Panel members gave oral or written feedback on both the framework and methodology. Feedback from one-on-one meetings (*N* = 3) was recorded and transcribed. Clarifications that were asked via email, were also included as part of the data.

### Data analysis

**Figure 1.**
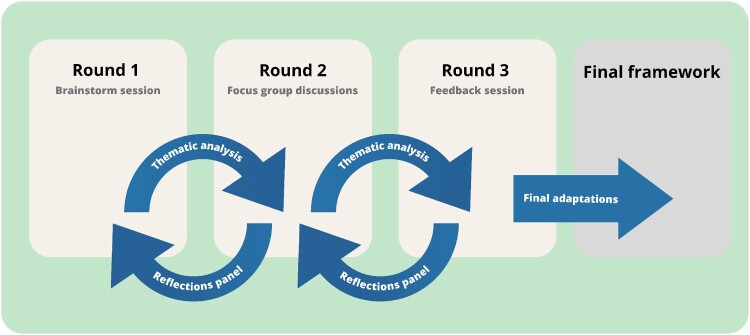
Overview analytical process

Focus group discussions were recorded, transcribed and analysed by the first author. Throughout the process, detailed field notes were maintained to track assumptions, reflections, and thoughts as a means of documentation. The iterative and reflexive approach of Braun and Clarke allowed for identifying themes inductively.^37,38^ The thematic analysis in this study was conducted in three iterative rounds, each involving systematic procedures for coding, theme generation, panel member feedback, and theme refinement to ensure a comprehensive and panel member-validated analysis. Figure 1 presents an overview of this process.

After Round 1, codes were generated based on written responses obtained from the panel members. These initial codes were thematically organised and subsequently reviewed with the panel members during Round 2 to ensure accuracy and alignment with their perspectives. Panel members’ comments during this phase contributed to the inclusion of additional codes and the formation of overarching themes for the data of Round 1.

After Round 2, all transcripts were thoroughly analysed using NVivo software, and an inductive coding approach was employed to identify patterns and generate initial codes from the data. Codes extracted from multiple transcripts were collated and grouped to form overarching themes. To ensure fidelity to the data, transcripts underwent multiple readings, and themes were continually refined to encompass all pertinent codes and accurately summarise the diversity of perspectives present in the dataset. The generated themes were then verified with panel members during Round 3.

In Round 3, written comments and transcripts were coded and grouped into overarching themes.

Panel members were actively engaged in reviewing the preliminary framework and paper, drafted by the first author, ensuring that the identified themes in all rounds resonated with their experiences and accurately reflected the breadth of perspectives expressed.

### Ethics

The study received ethical approval from the Ethical Committee of Ghent University Hospital (Approval code: THE-2022-0132) on 23/08/2022. Panel members participated in individual online introduction sessions prior to participation, where they were presented with the informed consent form and given the opportunity to ask questions before signing. Withdrawal from the research process was possible at any time.

Confidentiality measures included disclosure of email addresses only with consent, pseudonymisation, and deletion of discussion recordings post-transcription. Co-authorship was offered to all panel members due to the co-creative design. We chose not to collect detailed demographic information from the panel members to avoid making anyone uncomfortable. Some participants voluntarily shared personal details, but we will report this information cautiously to protect their privacy. Additionally, any sensitive quotes were rephrased according to the participants’ preferences to ensure their comfort and confidentiality.

## Results

A total of 15 panel members agreed to participate in this Delphi study. Based on the personal information provided during the discussions, we can say the panel included people with different gender identities (man, women, genderqueer), sexual orientations (heterosexual, bisexual, pansexual, queer), relationship forms (monogamous, polyamorous), and different countries of origin (Belgian, British, German, Romanian, Georgian, Italian-Filipino, Colombian, Nepalese, Ghanaian, Kenyan, Malawian, Kazakh, Turkmen).

### Round 1: Asynchronous written brainstorm

A total of 11 panel members contributed to the brainstorm session. This number was derived from Padlet, which can track the number of contributors. Because of the anonymity of the responses in this round, it is unclear which panel members contributed. Annexe 3 provides an overview of responses to all the questions. In the following section, responses are synthesised into key topics.

#### Defining and assessing sexual well-being

Panel members were asked what words they would want to see in a definition of sexual well-being. An analysis of these words showed these can be grouped into five categories: (**1) Consent**, **feeling comfortable, accepted and safe**: Responses highlight the importance of sexual self-esteem, the ability to consent, and feeling respected within society. (**2) Pleasure and fun**: Responses emphasise that sexual experiences should be pleasurable, fulfilling, and enjoyable. (**3) Freedom and agency**: Responses emphasise the importance of having the freedom to express one’s sexual orientation and enjoy one’s sexual life, and being in control over one’s body and sexual choices. (**4) Knowledge and awareness**: Responses emphasise the importance of awareness and understanding of sexual pleasure, as well as knowledge about safe sex practices. (**5) Holistic, relating to mental, physical and social health:** Responses highlight the importance of an overall healthy and happy state, including psychological happiness, physical health, a sense of connection to one’s sexual identity, and fulfilment in one’s sexual life. Panel members also shared what questions they would ask young people of their community to explore their sexual well-being. Based on this question, the following additional topics could be identified: (**6) Communication:** Responses highlight the importance of comfort and openness in discussing sexuality with various individuals, including partners and peers. They also address the ability to communicate and negotiate safe sex practices effectively. (**7) The state of sexual health and rights in society,** such as access to abortion, sexuality education and contraception; and (**8) Attitudes and norms** of, for example, service providers, and young people themselves, towards sexuality of young people. The exact responses and how they fit in the different categories are presented in Table 1.

**Table 1.** Results brainstorm

Defining sexual well-being		Assessing sexual well-being	
Sexual well-being is about consent, feeling comfortable, accepted and safe	Comfortable Feeling comfortable in their own sexual life and health Having positive views about one’s self sexually Respect Safety Safe Consent Acceptance Inclusion	Sexual well-being is about consent, feeling comfortable, accepted and safe	Are you feeling comfortable with your own sexual well-being? What opinions do you have about yourself and body sexually? Do you feel safe in your community to express your sexual identity? Do you understand and practice consent? How do you express consent to sex?
Sexual well-being relates to pleasure and fun	Being able to achieve satisfaction from one’s sexual life Experience of sexual pleasure Pleasurable Fulfilling Enjoyable	Sexual well-being relates to pleasure and fun	Are you experiencing sexual pleasure and satisfaction whenever you want it? Are you satisfied by your sexual life? Do you enjoy your sexual life? Are you experiencing pleasure?
Sexual well-being relates to freedoms and agency	Empowerment Freedom to express sexual orientation Bodily autonomy Free	Sexual well-being relates to freedoms and agency	Do you feel empowered to experience pleasure? Are you able to enjoy pleasure and intimacy without pressure from society, influence of stereotypes, stigma, and discrimination? Are you able to freely choose your intimate partner?
Sexual well-being relates to knowledge and awareness	Awareness Understanding of sexual pleasure Knowledge about safe sex practices with the skill to execute/negotiate about it Good-quality information	Sexual well-being relates to knowledge and awareness, and safe sex practices	Do you know the signs of any STI? Do you know there is something called safe abortion? Do you notice changes when it comes to your sexual health? Do you have any idea of what sexual well-being is? What can cause you to miss your period? What is your level of understanding on sexuality education? Have you heard of “safe period” before? Do you know how to track your menstrual cycle? How often do you take STI tests? Will you consider practicing safe sex? How do you prevent pregnancy? What do you think of safe sex? Do you practice it? Do you know what sexual act is and how does it conducted?
Sexual well-being is a holistic concept and relates to mental, physical and social health	Complete/whole, healthy, mental, social relations, body, emotional Complete, state of health, reproductive system, functions Healthy physically and internally (psychologically), or happy in connection to sexuality Complete natural wellness, beyond physical look, emotional connection with our sexual orientation A state of one’s sexuality in relationship to his or her Social, physical and emotional ability.	Sexual well-being relates to the state of sexual health in society e.g. accessibility of sexual and reproductive health services	What are the indicators for abuse, STI rates? Is abortion legal? Do you have access to sex education? Are you able to seek help? Do you need help to find somebody to talk to? Are you able to access the contraception of your choice? Where do you find information? What information do you miss? How do you fill in your missing information?
Other	Absence of disease	Sexual well-being relates to attitudes and norms	What are your views on service providers introducing contraceptives to young people? Do you feel it’s appropriate to know anything about your sexual well-being? Do you see sexual well-being to be of any importance?
		Other	How often do you engage in sexual activity? Have you ever had a sexual relationship with anyone? Have you at all touched, kissed or hugged the opposite sex? Firstly, I will ask them: Have you scored less in exams before? What did you do? I will definitely get someone to say “I cried or I was worried” then let them know that worrying and crying is part of your sexual well-being in relation to psychology. I will if anyone is talking to a friend here or how you feel when your teacher pairs you with the opposite sex. The answers will also let me know how well their physical relation is

#### Sexual well-being versus sexual health

Panel members’ discussion about the relationship between sexual health and well-being, revealed diverse perspectives. Some view sexual health as encompassing well-being, while others see well-being as broader, with sexual health focusing more on biological aspects. Yet, some perceive both concepts as similar.

#### Sexual well-being and intersectionality

Panel members considered how their intersectional identities impacted their understanding of sexual well-being. Almost all respondents acknowledged the influence of factors like gender, sexual identity, and upbringing on their perception, although some seemed to focus more on how their identities like gender, health status, and personality, influenced their state of well-being rather than their conceptualisation of it.

#### Defining sexual activity

Finally, panel members shared their definition of sexual activity. Most panel members acknowledged that sexual activity is not limited to penis-in-vagina sex, and should be defined holistically, encompassing different kinds of acts (e.g. kissing, hugging, fondling, masturbation, etc.), and different kinds of elements (e.g. pleasure, intimacy, arousal, etc.). Some panel members stated people should be free to have their own definition of sexual activity.

#### Conclusion round 1

Based on the responses of the first round, it seems sexual well-being is a concept that should be defined and measured in a broad sense. It entails elements of consent, safety, acceptance, pleasure, and communication. It relates to having knowledge, self-awareness and self-esteem. Additionally, broader societal aspects, such as access to SRHR services and social norms, appear to be relevant for the concept. While panel members did not necessarily agree on how sexual well-being and sexual health exactly relate to each other, they did agree the two concepts are closely interrelated. Most panel members acknowledged the relevance of intersectionality, either as an influence on the conceptualisation of sexual well-being, or as a factor influencing their state of sexual well-being. Sexual activity should not be limited to penetrative sex, but should be defined broadly, leaving room for different interpretations.

### Round 2: Focus group discussions

In total, 10 panel members participated in the online panel discussions. Session 1 involved three panel members (PM1, PM2, PM3), session 2 involved four panel members (PM4, PM5, PM6, PM7), and session 3 involved three panel member (PM8, PM9, PM10). One panel member (PM11) provided written input on the summary of the three discussions.

In the three discussions, panel members reflected on previous literature and on their own brainstorm responses. To the panellists’ surprise, there were a lot of similarities in the responses in the brainstorm. Panel members expected more variety in the interpretation of sexual well-being because of their different contexts. One panel member commented that the common background, as young professionals in the field of SRHR work, might have contributed to these similarities.

Five themes were derived from these discussions that appeared to be key for the conceptualisation of sexual well-being: Sexual well-being is not a norm; Sexual well-being is part of a complex system; Sexual well-being relates to opportunities and liberties; Sexual well-being is a privilege; The state has a responsibility in sexual well-being.

#### Sexual well-being is not a norm

The literature sometimes defines sexual well-being as “the individual experience of their sexual lives”.^35,39,40^ Panel members agreed that incorporating a subjective element in the concept of sexual well-being that takes into account different needs and goals, is relevant as it might look different for individuals with different intersectional identities.

*“I will try to make a comparison with how people, define sex and gender. […] I think that sexual well-being could be on the same page as gender in my comparison. It’s more like a construction of how you identify it yourself, how you identify, your sexuality and how you feel comfortable with that.”* (PM6)

Different individuals, with different sexual orientations, in different relationship forms, might have different needs and desires in terms of what they want to gain from a (sexual) relationship. It is important to acknowledge that *“There are probably a hundred other reasons why someone might have sex or not”* (PM1).

Panel members stated some indicators that are currently used in the literature fail to acknowledge the subjectivity of sexual well-being, and make it a normative concept. For example, when using the sexual function or frequency of sexual activity as a proxy for sexual well-being, it doesn’t leave room for the fact that sex can look different for different individuals, without being “abnormal”. Some panel members noted this can be avoided by asking if people are satisfied with their sexual function or sexual frequency, while acknowledging that it is okay to be satisfied if they are not having sex at all.

*“A lot of the definitions can make it seem as if we are not doing ‘ABC’, then there’s something wrong with you.”* (PM3)

Diversifying the idea of what sexual activity means, and adding an intersectional lens to it, was reckoned to be essential to avoid normatively measuring sexual well-being. Sexual activity might look different for people with disabilities, people with different sexual orientations, people with certain religious values, or survivors of sexual violence that had to redefine what sexual activity is to be able to gain pleasure from it. It can look different for any individual depending on their preferences, without it being abnormal. Within sexual well-being, the only element of sexual activity that has to be pre-defined, is that it needs to be consensual.

*“I think that most of the times, sexual satisfaction is only connected with, penetrative sex. So then it’s a very limited view of like, ‘are you having this, then you are satisfied or not’. So maybe this is why it’s also the same case for people with disabilities, because you don’t have to get satisfaction only from that.”* (PM9)

#### Sexual well-being is part of a complex system

Adding more subjectivity does not suffice to capture the concept of sexual well-being. Panel members agreed that limiting the conceptualisation of sexual well-being to subjective well-being fails to acknowledge the complexity of the societal system that individuals live in. For example, individuals might be satisfied because they just don’t know better.

*“When subjectivity takes the central role, then it becomes ‘what I think is right, what you think is right’. And it may not be. What I think is right may be based on how well-informed I am.”* (PM3)

They also feel that societal norms feed into the conceptualisation of the sexual well-being of the individual. Societies have strong visions on what sex lives should look like. In some societies, sex is considered to only have a reproductive purpose, while in other societies sex is central to having a good marriage or a happy life.

*“We are also very much socialized into thinking that we are very sexual beings, and we should always be like, you know, it’s a very instinctual thing when a lot of the time it isn’t.”* (PM4)

*“When it comes to sexual well-being, a lot of the times, it’s not something that readily comes to mind or something that is really important. […] Because you’re a young person it’s believed that sex shouldn’t be a focus for you.”* (PM3)

Harmful norms are reflected in and spread through pornography, media, peers, and even in certain sexual education programmes and internalised by individuals, which raises the question whether individuals are ever truly free in choosing their preferences.

*“I feel like the system that we live in, like the patriarchy and capitalism, really do influence the way that we think about sex. For example, there’s pressure for men to have sex. And there’s also really a bad spot for women to talk about sex freely. So I do think that it affects all the genders.”* (PM9)

#### Sexual well-being relates to opportunities and freedoms

Being able to pursue one’s preferences, in terms of choosing a partner and the kinds of sexual activity one engages in, is a privilege for individuals who live in environments that enable these subjective decisions.

*“Subjective well-being and of course capability are both equally important on their own. You can very well have necessities and desires, but also not be able to act on those necessities and desires, which then if you don’t have the capability, then it doesn’t really suffice in that sense.”* (PM4)

Sexual well-being is about having control over one’s sexual decisions and experiences. Individuals might be coerced into certain activities by their environment, limiting the ability to make subjective decisions that are truly theirs. This also means that individuals are not always able to truly consent.

*“When we speak of the freedom from pressure, to really include pressure that is felt by social norms, […], if we are to create a framework for sexual well-being, I think that is a very important aspect because, you’re not fully liberated either if you’re still pressured by these social norms.”* (PM4)

Therefore, the panel members feel it is important to take a broad approach and understand the capabilities or freedoms and opportunities of individuals, and how these shape subjective well-being.

*“I like the idea of the ability to access sexual well-being or to access a full sex life instead of, what that actually is, if that makes sense.”* (PM1)

A diverse set of interrelated factors were identified by the panel members. Individuals need to be able to be comfortable with who they are. They need self-esteem, bodily autonomy, the ability to explore, and the ability to communicate about their needs, the ability to have and accept pleasure. Furthermore, they also require the ability to make informed choices, and the ability to have safe sex. They require self-awareness, about their needs, and what makes them feel comfortable or what triggers trauma and self-awareness about their health status, and how it relates to their sexual health. Most importantly, they require the ability to consent.

Many of these factors are interlinked. It was for example discussed how bullying might have a significant impact on a person’s self-esteem and body image, limiting their ability to be comfortable with themselves and, therefore, their ability to explore themselves sexually. Having a level of comfort, which also means comfort with one’s sexual identity, influences one’s ability to communicate with partners and consent.

*“There are a lot of people who have engaged in sexual activities, but because they don’t know or they are not comfortable with who they are, or what will actually make them happy, or what is pleasurable to them, they are forced to succumb.”* (PM3)

Sexual well-being therefore requires general well-being in society. Comforting and safe environments create the ability to communicate, both with partners, and with other people in the community. For example, in more conservative societies, individuals might not feel as comfortable communicating their preferences to their sexual partner, or to talk about sex in their community because of sociocultural taboos.

*“In my country, because people are quite conservative, not all, but most people think about it as like one of the duties. So they don’t like, for example, after the sex talk about it, if they like it or that maybe there was something that they wanted to have next time. So it’s not that much common to talk about this stuff.”* (PM8)

Access to SRHR services, and quality information through sexuality education and modern media, enables individuals to be comfortable and make personal choices that are informed. It enables individuals to be self-aware about their sexual health status, their needs and preferences, and the ability to manage their sexual health.

*“So for example for me feeling comfortable, it’s also like feeling comfortable with the rights that you have for example for contraceptive methods and things like this. So it does include other things.”* (PM9)

*“[good quality information] is for me the most important because I think like if everybody has the access to good information or knows where to find it or who to talk to that, you get the chance to create a better sexual being for yourself.”* (PM5)

#### Sexual well-being is a privilege

Enabling environments are more accessible for certain groups than others, making sexual well-being a privilege. Therefore, panel members feel it is important to also add an intersectional lens to measuring sexual well-being, which is lacking in the current measurements of sexual well-being. An individual’s freedoms and opportunities are affected by the way society looks at their sexual lives, which highly depends on the intersectional identity of a person. In most societies, sexuality, and the conversations around this, remain taboo, especially for groups such as young people, women, LGBTQIA+, persons living with a disability, ethnic and religious minorities, and people on the move. This influences the possibility to talk about it, the possibility to have relationships or sex lives, to accept pleasure, to have availability of suitable information and services, and thus people’s ability to achieve sexual well-being.

*“So how the human rights ecosystem of where you are, affects how you get to experience your sexual well-being. If you are unfortunate, then you are in a very patriarchal system where people are rather making efforts to reduce the rights of certain genders. Say you’re a woman or say you belong to the LGBTQI+ community, that then gets to affect how you get to experience your sexuality, how your day-to-day living or, the perpetuation of your sexual being then gets to be.”* (PM3)

Table 2 provides an overview of how the panel members felt societal norms affect different groups’ access to sexual well-being.

**Table 2.** Sexual well-being is a privilege

Women	
Women's sexuality is considered to be shameful	“There is more freedom for men to have an active sex life, but at the same time for women, it’s like something that you should be ashamed of.” (PM9)
	“I know, there is also one group on Facebook, it’s a space for female talks. And lots of girls, what they write nowadays, especially in terms of like sexual pleasure, is that like they cannot get rid of this thought that they’re doing something bad. And then it of course changes everything, the way you understand your sexual well-being or pleasure and this concept.” (PM2)
Women’s pleasure is less important	“I think also relating to pleasure, I think, for a lot of people having grown up, in conservative societies and not being able to explore your sexuality and you like have, this idea that in sex, especially when you watch non-feminist porn, that the woman is just there and your pleasure comes second in these heterosexual relationships.” (PM4)
Women’s pain is normalised	“And I think another interesting thing that I was just thinking about is touching on the idea of lubrication and kind of like, where social norms come into that. Where there might be pressure for it to be painful because of porn, and because of this idea that a woman’s got to have a really tight vagina.” (PM1)
Higher contraceptive burden for women	“So the double standards, for example, for men pregnancy is not an issue. But for women it is. So you have to take more steps into contraceptive issues and that also affects your body in a kind of way. So it is different then, and I feel like the society affects, most of them. If I cannot access an abortion, then it does affect my body. If I cannot contest the rape, then it affects my body.” (PM9)
**LGBTQIA+**	
Have less access to relationships, and SRH services	"(Talking about being queer in country where it is not allowed) … You don’t have like the availability to have a relationship or even like discuss with your doctor about it … Or like contraceptive methods for same sex relationships, they aren’t even advertised. Most people don’t even know about it because there is the whole idea that you don’t need to have it if you have same-sex intercourse.” (PM9)
Have less access to information	“There’s so many norms and I think we have to try to see that there are other options and also give information about the other options. There are, like one of the panel members said about polyamory. I find it really sad that nobody really knows about it. While, it is also a super valid choice to make. And I think there should be more information about it. But I think that’s maybe also a thing here. How easily do you have access to information? Because I think that’s sometimes really hard depending of your situation or your surroundings that you don’t have access to information and then it’s really hard to build your personality and stuff.” (PM5)
**People living with disabilities**	
Have less access to suitable information	“I think I don’t really see it, but please correct me if I’m wrong, but like the ability and like accessibility to have it. So I’m thinking mostly of people with disabilities for example. How easy it is for them to express this in a relationship or how is it is for them to access it? Or how do they get the knowledge about this and stuff like this you know?” (PM 9)
**Ethnic minorities**	
Have less access to suitable information and services	“In the context of my country, there are a lot of ethnic groups. So in that case, some things like the personal abuse or social norms or the communication or the access of sexual reproduction health services reflects in the multiple or the serial inequalities that we have. But also reflects that in the case of these ethnic groups, they have their own construction, have their own definitions and understandings. While we think that maybe this is incorrect in the way that they living their sexual well-being, it could be correct for them.” (PM6)
**Sex workers**	
Have less access to sexual well-being because of stigmatisation	“So I feel like it’s important to mention it. That it does exist, and it’s like a real profession and you still have to be aware of, like all the risks that involve like having this profession. Because I feel like they need it the most, but they don’t have access to it.” (PM9)
**Young people**	
Have less access to comprehensive information	“There does feel like this barrier where, under 16 you can’t tell them yeah you can have pleasure during sex. You’re not allowed to mention that at all because you’re supposed to be teaching them that they can’t be having sex at all because of the legal age is 16.” (PM1)

#### The state has a role in sexual well-being

The state has a responsibility in creating these enabling environments by enforcing laws, policies, addressing the taboo, ensuring sexual education in curricula and sexual health provisions for everyone, including for young people. One participant emphasised the impact of unprosecuted rape cases on sexual well-being, highlighting how trauma is intensified and the ability to give consent is compromised when perpetrators are not held accountable. It is crucial to prioritise these matters on the youth agenda and actively involve young people in these discussions.

*“If there are national policies in place that makes it easier for young people to access sexual and reproductive health services, that would eventually help in improving their sexual well-being. But if there are policies in place where there’s certain sexual orientations or being from a queer community or whatever is not legal, that is suppressing the sexual well-being in a broader sense.”* (PM7)

#### Conclusion round 2

During the second round, the panel members elaborated on the responses of the first round, and reflected on the existing literature. Panel members confirmed that sexual well-being is a concept that has to be defined and measured broadly, with attention to intersectionality. This means acknowledging that different individuals have different desires and goals, and not everyone is equally capable to define and obtain their desires and goals. Similarly to the first round, consent, comfort, safety, acceptance, pleasure, communication, knowledge, self-awareness, freedom, agency, access to SRHR services, and social norms, appear to be central to the concept of sexual well-being. Panel members also emphasised the importance of recognising the state’s role in sexual well-being.

### Round 3: Discussion of the draft conceptual framework

Based on the recommendations and input from the different panel members, a draft definition and framework was proposed by the lead researcher. Twelve panel members provided feedback on the definition, and the framework (nine in writing and three in an individual interview). Two of the panel members who gave oral feedback, did not participate in the live panel discussions (PM11, PM12).

In general, panel members were satisfied with the extensiveness of the framework. Most requests for changes focused on rewording, clarifying, or accentuating certain elements or making some visual changes. One of the panel members emphasised the fact that more voices needed to be included to further refine the framework.

### Consensus of the panel

**Figure 2.**
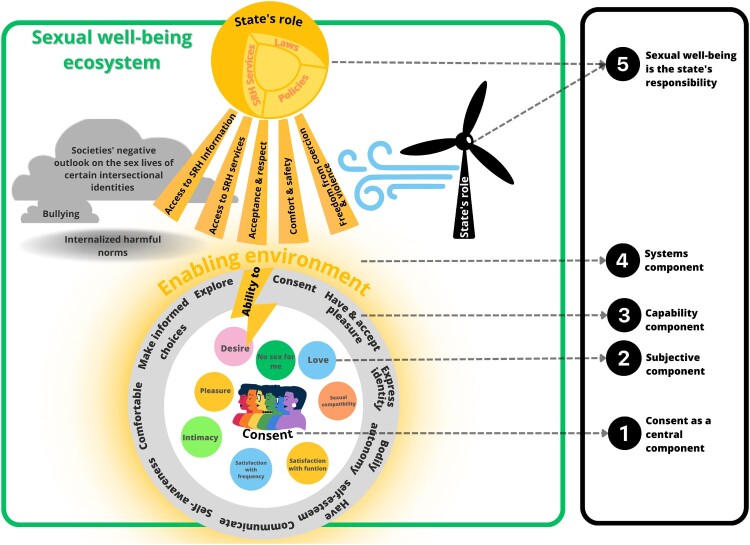
Framework for sexual well-being

The panel reached consensus on a framework (Figure 2) and definition for sexual well-being:

*“Sexual well-being is the ability of an individual, as a part of a complex societal system, to understand and fulfil the needs and desires of their sexual life. This means that the state has a responsibility in creating enabling environments, where individuals from all intersectional identities are empowered and feel comfortable and accepted for who they are, and have the freedom to consent and make decisions about their sexual lives. Sexual well-being therefore also means having access to sexual and reproductive services, information, access to comfort and safety, acceptance and respect, and freedom from coercion and violence.”*

Figure 2 provides a visual overview of the framework. The framework can be broken down into five different components. These components are also highlighted in Figure 2. A more detailed visual presentation and narrative are presented in Annexe 4.

#### Consent as a central component

Although the framework leaves space for diverse interpretations, consent was recognised to be a non-negotiable component of the sexual well-being framework. This underscores that coercive sexual activities are fundamentally incompatible with the framework.

#### Subjective component

This part of the framework acknowledges the subjective nature of sexual well-being, recognising that its meaning can vary among individuals and communities. For some, sexual well-being might involve finding intimacy in a relationship, while for others, it may mean choosing not to engage in sexual activity. The relative importance of different aspects of sexual well-being (e.g. desire, function, pleasure, intimacy, love) can differ between individuals and can shift over time. Definitions of “sexual activity” can vary and should not be limited to a definition of penetrative intercourse. Similarly, “intimacy” should not be limited to physical intimacy. This part of the framework allows individuals, such as queer people and those with disabilities, to redefine sexual well-being for themselves without being seen as abnormal. This part of the framework elicits continuous discussions about the concept of sexual well-being.

#### Capabilities component

This part of the framework recognises that people may not always have the opportunity to fully grasp and define what sexual well-being means for them in a manner that rightfully honours their needs and experiences. Therefore, we dedicated this part of the framework to some fundamental dimensions. These include the ability to consent, explore, make informed decisions, have bodily autonomy, have self-awareness regarding sexual health and what makes someone feel comfortable. For young people who have experienced trauma, self-awareness includes being trauma-informed, meaning they have an understanding of how past experiences may shape their sexual well-being. Additionally, the framework includes the ability to experience and accept pleasure, communicate, feel comfortable before, during, and after sexual activity, and have self-esteem.

#### Systems component

This part of the framework accounts for the complex system around the individual. For an individual to be able to grow in their capabilities, understand and pursue their personal desires and preferences, enabling and just environments need to be in place. Therefore, sexual well-being also means having sexual rights such as access to SRHR information and services, having access to acceptance and respect, comfort and safety, and freedom from coercion and violence within society. Unequal access to these sexual rights creates inequalities in sexual well-being.

#### State’s responsibility component

This part of the framework emphasises the responsibility of the state in sexual well-being. The state must take proactive steps to create enabling environments, ensuring equal access to sexual rights for all. First of all, this can be achieved through the provision of laws, policies and infrastructure. Some examples are anti-discriminatory laws, abortion laws, youth-inclusive sexual health policies. Secondly, the state should rectify societal biases that devalue and stigmatise the sexual lives of certain groups and therefore limit their sexual rights. These groups include, among others, women, youth, LGBTQIA+, individuals with disabilities, and sex workers.

## Discussion

The aim of this study was to develop a preliminary culturally sensitive and inclusive conceptual framework for sexual well-being. In this framework sexual well-being is conceptualised from a rights-based, intersectional, systems perspective. This means that the subjective nature of sexual well-being is acknowledged and the right of individuals and communities to contribute to discussions on sexual well-being is recognised. Additionally, it is recognised that all individuals are embedded in cultural value networks impacting their beliefs and desires regarding SRHR and that many are living in a system with unequal access to sexual rights, so that they may not always be in a position to identify, express, and obtain their needs and desires in a rightful manner. Sexual well-being is therefore defined as having fundamental capabilities such as the ability to consent, make informed decisions, and communicate.

The framework is in line with Lorimer et al.’s ideas.^18,19^ Following the reasoning of the “Capabilities approach” as introduced by Amartya Sen and Martha Nussbaum,^41^ Lorimer et al. noted that people living in oppression might adapt and thus report sexual well-being. To address this key limitation of subjective assessments, they concluded that sexual well-being should therefore also include people’s ability to live the lives they value, and assess opportunities and liberties.^18,19^ Our definition and framework take this into account and highlight the importance of a “sexual well-being ecosystem”. This encompasses inclusive SRHR policies, laws and infrastructure that ensure access to information, services, acceptance and respect, and comfort, safety and freedom from violence and coercion for every individual. This enables individuals to pursue their desires and preferences, consent and make informed choices. Following this reasoning, one could say that today many populations lack high sexual well-being, as different states have been reducing the sexual rights of their citizens.^42,43^ This is particularly true for women, young people, LGBTQIA+, people living with disabilities, ethnic minority groups, and people living in poverty.^44^ These groups have less access to SRHR services, education, and are more often exposed to violence and harmful norms. Also in the online world, particular groups, such as young women and gender and sexual minorities, are more frequently victimised.^45^ Young people increasingly seek online for information, which is not always correct and can contain harmful messages.^46^ These facts demonstrate the importance of extending the sexual well-being ecosystem to the online world.

The framework proposed by our panel aligns well with other models of sexual well-being. For example, the key competencies identified in Kågesten and van Reeuwijk’s competency-based framework (e.g. sexual literacy, interpersonal relationship skills, consent), as well as the importance of both personal (e.g. self-esteem, identity) and relational (e.g. safety and security) sexual well-being, are consistent with the panel’s framework.^15^ The primary difference is that, while they view the resources, agency, and opportunities that enable adolescents to apply competencies as separate from sexual well-being, the panel considers these elements to be an inherent part of it. De Meyer et al.’s idea of social (sexual) well-being is also reflected in the panel’s framework, particularly in how societal norms about the sexual lives of certain groups of people, affect access to sexual well-being.^22^ In Mitchell et al.’s framework, some of the domains of sexual well-being – such as self-determination, comfort with sexuality, sexual respect, safety, and self-esteem – are consistent with the panel’s framework, as well as the connection between sexual well-being, sexual health, pleasure, and justice.^20^ The main distinction lies in the panel’s perspective, where they view sexual pleasure, or at the very least the capability for pleasure, as an integral aspect of sexual well-being. Overall, while there are some nuanced differences, the panel’s framework integrates many core ideas from these existing models, offering an integrated perspective on sexual well-being that is grounded in their experiences.

**Figure 3.**
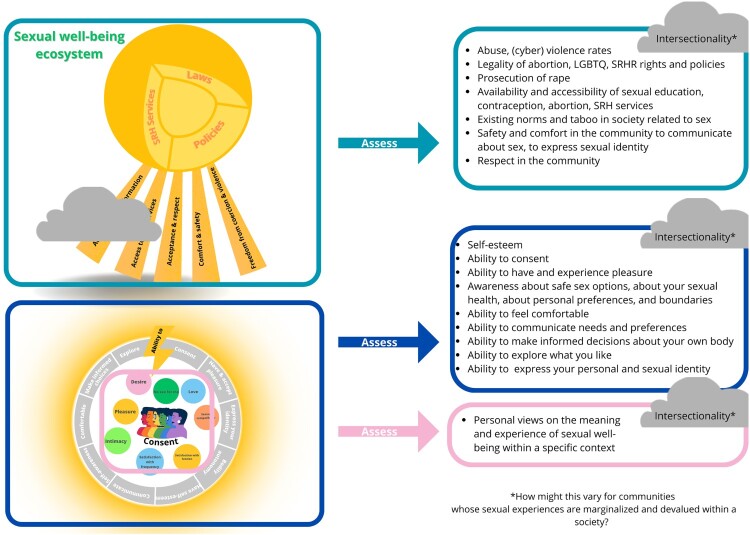
Practical application of framework

The framework identifies key domains that are valuable for assessing sexual well-being of young people, and guides the selection of appropriate scales or indicators. We see complementary potential between Mitchell et al.’s Natsal-SW scale and the domains identified in this framework, with opportunities to incorporate some scale items into the capability component of this framework.^21^ However, the subjective component of the panel’s framework encourages continuous discussions with local communities, as they view ongoing dialogues as essential to rights-based sexual well-being work. To adapt the Natsal-SW to different contexts and specific sub-groups, such as young people, we would therefore recommend using bottom-up participatory approaches in validation processes. This approach would allow measurement of the concept while also acknowledging its subjectivity. Additionally, to address the systems component of sexual well-being, we recommend that researchers use analytical techniques to assess intersectional differences and inequities in capabilities, as well as access to enabling environments. Figure 3 demonstrates how this view can be translated in a more practical manner, to encourage researchers to measure sexual well-being, notwithstanding its complexity. By targeting a range of capabilities and positive subjective experiences, with careful attention to intersectional differences, researchers can conduct comprehensive assessments that account for the complex social contexts young people navigate, shaped by systemic injustices.

Our study had several strengths. The panel was diverse, including individuals from different countries, genders, and sexual identities. Multiple rounds of data collection allowed panel members to deepen their understanding of sexual well-being, and actively engaged them in data analysis and priority-setting of the messages they wanted to share. Multiple formats of data collection allowed panel members with different preferences of learning and sharing knowledge, to contribute. The modified Delphi methodology facilitated consensus while allowing for discussions that would not have been possible with a traditional Delphi design. We agree with Guzys et al. that a constructivist framework is likely more appropriate for the Delphi methodology. We therefore recommend that researchers consider integrating constructivist principles when using this methodology.^29^

While the methodology offered opportunities for a culturally sensitive and inclusive framework for sexual well-being, it is important to acknowledge its limitations. The design’s flexibility may be less rigorous compared to traditional research designs. The participatory design and the inclusion of panel members as co-authors limited our ability to provide detailed demographic information about the panel, as we aimed to protect their privacy. As a result, it becomes challenging to contextualise the quotes and framework within specific demographic perspectives. The panel, although diverse in terms of gender and sexual orientations, as well as geographical diversity, was not representative for every young person worldwide. Adolescents, sex workers, people living with disabilities, transgender people, people identifying as asexual, and people without professional or educational background in SRHR, could have shared additional insights for a more comprehensive understanding of sexual well-being. While the close alignment of our framework with earlier models is encouraging, as it shows that contemporary perspectives of diverse young adults resonate with decades of research that informed previous models, it does not imply that we have definitively answered how sexual well-being should be conceptualised. We describe it as a “preliminary” framework, representing an initial effort to incorporate more bottom-up perspectives. Given that the panel members were already professionally involved in SRHR, the narratives and vocabulary they used likely aligned with their existing experiences in the field. To create a truly culturally sensitive and inclusive framework, further efforts are needed to expand beyond these perspectives, incorporating a wider range of voices and moving beyond the dominant narratives and vocabulary within the professional SRHR landscape.

A critical consideration involves questioning whether a framework based on the capability approach can genuinely embrace inclusivity and cultural sensitivity, as this approach has been criticised for potentially upholding patriarchal and imperialistic facets in its application.^47,48^ Our framework acknowledges individuals’ agency to shape their own understanding of sexual well-being, but also underscores the importance of a sexual well-being ecosystem that includes specific capabilities. While the former aspect facilitates the inclusion of indigenous and local values, the latter might be considered as a way of withholding power. It is important to engage in further discussions to understand whether and how we can balance these approaches to ensure cultural sensitivity, and hold states accountable for warranting and assessing sexual well-being in an equitable way, without reinforcing neocolonial ideologies.

The limitations identified do not diminish the importance of the contributions made by this work. First, we offer a meaningful contribution to the ongoing exploration and refinement of sexual well-being frameworks, adding the perspectives of young adults. Second, we piloted an interesting method, centred on integrating the voices of youth, which offers a promising initial approach that can be further tailored and expanded upon in future research endeavours, especially to engage with adolescents, and more marginalised and vulnerable young populations. Finally, this paper highlighted the essential responsibility states can play in ensuring sexual well-being of their young population.

As a next step, it is critical for governments, researchers, and policy-makers to uphold their responsibility, prioritise young people’s voices and needs, particularly those of minority groups, and recognise every young person’s fundamental right to sexual well-being. The Scottish government has set an example by integrating sexual well-being into their sexual health action plan and population surveys.^49^ They acknowledge the diverse actions required for sexual well-being, including service provision, education, information, gender equality, sexual expression, mental health, addressing gender-based violence, and justice. Their initiative serves as an example for other countries to advance sexual well-being for all young people.

## Conclusion

Our framework provides a good starting point for a comprehensive theoretical understanding of the concept of sexual well-being of young people. It indicates the importance of defining and measuring sexual well-being as a system, whilst paying attention to intersectional differences in its understanding. This conceptualisation can guide future research, policymaking and programming, towards more positive approaches.

## Supplementary Material

Annexes 1-4

## References

[1] WHO. Sexual health. Available from: https://www.who.int/health-topics/sexual-health#tab=tab_1

[2] Hadi M. Historical development of the global political agenda around sexual and reproductive health and rights: a literature review. Sex Reprod Healthc. 2017;12:64–69. doi:10.1016/j.srhc.2017.03.005 28477934

[3] Starrs AM, Anderson R. Definitions and debates: sexual health and sexual rights. Brown J World Aff. 2016;22(2):7–23.

[4] WHO. Sexual health and its linkages to reproductive health: an operational approach; 2017.

[5] WHO. Defining sexual health: report of a technical consultation on sexual health, 28–31 January 2002. Geneva: World Health Organisation; 2006.

[6] Wellings K, Johnson AM. Framing sexual health research: adopting a broader perspective. Lancet. 2013;382(9907):1759–171762. doi:10.1016/S0140-6736(13)62378-8 24286782

[7] Mattison C, Ateva E, De Bernis L, et al. Whose voice counts? Achieving better outcomes in global sexual and reproductive health and rights research. BMJ Glob Health. 2023;8(10):e012680. doi:10.1136/bmjgh-2023-012680 PMC10583075 37848270

[8] Starrs AM, Ezeh AC, Barker G, et al. Accelerate progress – sexual and reproductive health and rights for all: report of the Guttmacher– Lancet Commission. Lancet. 2018;391(10140):2642–2692. doi:10.1016/S0140-6736(18)30293-9 29753597

[9] Gruskin S, Kismödi E. A call for (renewed) commitment to sexual health, sexual rights, and sexual pleasure: a matter of health and well-being. Am J Public Health. 2020;110(2):159–160. doi:10.2105/AJPH.2019.305497 31913674 PMC6951372

[10] Landers S, Kapadia F. The public health of pleasure: going beyond disease prevention. Am Public Health Assoc. 2020;110:140–141. doi:10.2105/AJPH.2019.305495 PMC6951379 31913667

[11] Michielsen K, De Meyer S, Ivanova O, et al. Reorienting adolescent sexual and reproductive health research: reflections from an international conference. Reprod Health. 2015;13:1–5. doi:10.1186/s12978-016-0117-0 PMC4711048 26758038

[12] Pitts RA, Greene RE. Promoting positive sexual health. Am J Public Health. 2020;110(2):149–150. doi:10.2105/AJPH.2019.305336 31913675 PMC6951384

[13] Bond KT, Radix AE. Sexual health and well-being: a framework to guide care. Med Clin. 2024;108(2):241–255. doi:10.1016/j.mcna.2023.10.001 PMC12877561 38331477

[14] Harden KP. A sex-positive framework for research on adolescent sexuality. PerspectPsychol Sci. 2014;9(5):455–469. doi:10.1177/1745691614535934 26186753

[15] Kågesten A, van Reeuwijk M. Healthy sexuality development in adolescence: proposing a competency-based framework to inform programmes and research. Sex Reprod Health Matters. 2021;29(1):104–120. doi:10.1080/26410397.2021.1996116 PMC8725766 34937528

[16] Philpott A, Larsson G, Singh A, et al. How to navigate a Blindspot: pleasure in sexual and reproductive health and rights programming and research. Int J Sex Health. 2021;33(4):587–601. doi:10.1080/19317611.2021.1965690 38595783 PMC10903683

[17] WHO. Measuring sexual health: conceptual and practical considerations and related indicators. Geneva: World Health Organization; 2010.

[18] Lorimer K, DeAmicis L, Dalrymple J, et al. A rapid review of sexual wellbeing definitions and measures: should we now include sexual wellbeing freedom? J Sex Res. 2019;56(7):843–853. doi:10.1080/00224499.2019.1635565 31335208

[19] Lorimer K, Greco G, Lorgelly P. A new sexual wellbeing paradigm grounded in capability approach concepts of human flourishing and social justice. Cult Health Sex. 2023;25(10):1402–1417. doi:10.1080/13691058.2022.2158236 36565149

[20] Mitchell KR, Lewis R, O’Sullivan LF, et al. What is sexual wellbeing and why does it matter for public health? Lancet Public Health. 2021;6(8):e608–ee13. doi:10.1016/S2468-2667(21)00099-2 34166629 PMC7616985

[21] Mitchell KR, Palmer MJ, Lewis R, et al. Development and validation of a brief measure of sexual wellbeing for population surveys: the Natsal Sexual Wellbeing Measure (Natsal-SW). J Sex Res. 2023;60(1):1–11. doi:10.1080/00224499.2022.2035663 38127808

[22] De Meyer S, Jerves E, Cevallos-Neira A, et al. Which factors contribute to sexual well-being? A comparative study among 17 to 20 year old boys and girls in Belgium and Ecuador. Cult Health Sex. 2022;24(8):1122–1138. doi:10.1080/13691058.2021.1928288 34126851

[23] WHO. Intergenerational solidarity and adolescent wellbeing 2022 [cited 2024 June]. Available from: https://www.who.int/news/item/12-08-2022-intergenerational-solidarity-and-adolescent-wellbeing

[24] UN. United Nations: Sustainable Development Goals; Youth 2018. Available from: https://www.un.org/sustainabledevelopment/youth/

[25] Fletcher AJ, Marchildon GP. Using the Delphi method for qualitative, participatory action research in health leadership. Int J Qual Methods. 2014;13(1):1–18. doi:10.1177/160940691401300101

[26] Barrett D, Heale R. What are Delphi studies? Evid Based Nurs. 2020;23(3):68–69. doi:10.1136/ebnurs-2020-103303 32430290

[27] Shang Z. Use of Delphi in health sciences research: a narrative review. Medicine. 2023;102(7):e32829. doi:10.1097/MD.0000000000032829 36800594 PMC9936053

[28] Kivunja C, Kuyini AB. Understanding and applying research paradigms in educational contexts. Int J High Educ. 2017;6(5):26–41. doi:10.5430/ijhe.v6n5p26

[29] Guzys D, Dickson-Swift V, Kenny A, et al. Gadamerian philosophical hermeneutics as a useful methodological framework for the Delphi technique. Int J Qual Stud Health Well-Being. 2015;10(1):26291. doi:10.3402/qhw.v10.26291 25948132 PMC4422843

[30] Hesse-Biber SN. Feminist research practice: a primer. Thousand Oaks: Sage Publications; 2013.

[31] Lykke N. Feminist studies: a guide to intersectional theory, methodology and writing. New York: Routledge; 2010.

[32] De Villiers MR, De Villiers PJ, Kent AP. The Delphi technique in health sciences education research. Med Teach. 2005;27(7):639–643. doi:10.1080/13611260500069947 16332558

[33] Janssens F, Degomme O, Dewaele A, Peeters E. Conceptualizing a multi-dimensional framework for the evaluation of sexual well-being: a global Delphi study. 2022. https://lib.ugent.be/catalog/rug01:003157658

[34] Remmerie L, Michielsen K. Het concept seksueel welzijn: Hoe wordt seksueel welzijn gedefinieerd en gemeten in de wetenschappelijke literatuur? 2020. https://lib.ugent.be/catalog/rug01:002836700

[35] Verschuren JE, Geertzen JH, Enzlin P, et al. Sexual functioning and sexual well-being in people with a limb amputation: a cross-sectional study in The Netherlands. Disabil Rehabil. 2016;38(4):368–373. doi:10.3109/09638288.2015.1044029 26030200

[36] Mavhandu-Mudzusi AH. Impact of stigma and discrimination on sexual well-being of LGBTI students in a South African rural university. S Afr J High Educ. 2017;31(4):208–218.

[37] Braun V, Clarke V. Using thematic analysis in psychology. Qual Res Psychol. 2006;3(2):77–101. doi:10.1191/1478088706qp063oa

[38] Braun V, Clarke V. Reflecting on reflexive thematic analysis. Qual Res Sport Exerc Health. 2019;11(4):589–597. doi:10.1080/2159676X.2019.1628806

[39] Laumann EO, Paik A, Glasser DB, et al. A cross-national study of subjective sexual well-being among older women and men: findings from the Global Study of Sexual Attitudes and Behaviors. Arch Sex Behav. 2006;35:143–159. doi:10.1007/s10508-005-9005-3 16752118

[40] Muise A, Preyde M, Maitland SB, et al. Sexual identity and sexual well-being in female heterosexual university students. Arch Sex Behav. 2010;39:915–925. doi:10.1007/s10508-009-9492-8 19330438

[41] Robeyns I, Byskov MF. The capability approach. Stanford encyclopedia of philosophy. 2011. [Online] https://plato.stanford.edu/entries/capability-approach/

[42] Dupont J, Chollier M, Meštrović T, et al. Towards a transnational sexual health research and policy agenda: the European sexual medicine network Delphi study. Sex Res Soc Policy. 2022;19(4):1888–1903. doi:10.1007/s13178-022-00686-y

[43] Glenza J, Pengelly M, Levin S. Us supreme court overturns abortion rights, upending Roe v Wade. The Guardian. 2022;24.

[44] Sundewall J, Kaiser H. Sexual and reproductive health and rights: an essential element of universal health coverage. Background document for the Nairobi Summit on ICPD25-accelerating the promise. New York: UNFPA; 2019.

[45] Khosla R, Mishra V, Singh S. Sexual and reproductive health and rights and bodily autonomy in a digital world. Sex Reprod Health Matters. 2023;31(4):2269003. doi:10.1080/26410397.2023.2269003 37930349 PMC10629411

[46] UNESCO. Comprehensive sexuality education: for healthy, informed and empowered learners. Paris: UNESCO; 2023.

[47] Deneulin S. Perfectionism, paternalism and liberalism in Sen and Nussbaum’s capability approach. Rev Political Econ. 2002;14(4):497–518. doi:10.1080/0953825022000009924

[48] Vasbist L. Martha Nussbaum’s capabilities approach: perils and promises. J Indian Law Inst. 2010;52(2):230–266.

[49] Sexual health and blood borne virus action plan: 2023 to 2026. The Scottish Government, Directorate PH; 2023 28 November 2023. ISBN 9781835216590.

