# Supplementary material for: Towards an inclusive and culturally sensitive conceptualisation of sexual well-being of young people: preliminary framework development using a modified Delphi methodology
Source: Sex Reprod Health Matters. 2025 Mar 5;32(1):2474337. doi: 10.1080/26410397.2025.2474337 (PMC11983578; doi:10.1080/26410397.2025.2474337)

# Annex 1: Contacted organizations

| **Table 1: Contacted organizations** | | |
| --- | --- | --- |
| Aditi VZW  Advocates for youth  African Queer Youth Initiative  ARUS PELANGI  Ashanti Peru  China Youth Network  Choice for Youth & Sexuality*  Cuenca University  Disability-Inclusive Sexual Health Network*  ECA Youth Alliance Group  FOS Feminista  GALCK+*  HITOPS  *Organizations that responded | Indian ACES  International Federation for Spina Bifida and Hydrocephalus*  IYAFP  NOA (Nederlands organisatie aseksualiteit)*  OSSYR  Pacific LGBTQ Network  PANACEA  RHEP Foster Youth*  Sensoa*  Sexual Health Promotion and Education Research*  Thai Transgender Alliance Group  Trans Youth Equality | UNFPA APRO  UNFPA EECA  UNFPA Lacro  Women Deliver  Women’s Global Network for Reproductive Rights  World Association for Sexual Health*  YIELD Hub*  Youth Coalition*  Youth Tech Health*  YPEER Asia Pacific  YPEER General*  YPEER Kazakhstan*  YPEER Tajikistan  YPEER International Network*  YSAFE |

# Annex 2: Data collection tools

## Questions asked in round 1

1. If you would have to define “sexual well-being” for young people in your community, what words would you certainly want to see in the definition?
2. If you want to know how young people in your community are doing in terms of sexual well-being, what questions would you ask?
3. Sexual activity means different things to different people. If you were to define it, what aspects should the definition include?
4. What words would you use to describe your personal identity? Do you feel your identities influence how you interpret sexual well-being? How?
5. Do you feel there is a difference between 'sexual well-being' and 'sexual health'? How do you feel they relate to each other or how do you feel they differ?

## Questions asked in round 2

### Part 1: Reflecting on the brainstorming and the scientific literature.

1. Feedback on analysis of round 1
   1. Do you have any reflections about the brainstorming? How was it to answer the questions?
   2. Do you have any reflections/thoughts/additional questions after reading the responses of everyone?
   3. What are your most important takeaway messages?
2. Defining sexual well-being & defining sexual activity: You were asked to define both of these concepts.
   1. When going through the responses, are there things you find especially important? Are there things that you find troubling to see in a definition? Why?
   2. At this point, there is no official definition of sexual well-being. However, some authors have tried to define/describe this. Some of these descriptions are more general, some are more specific. What are your thoughts on these descriptions? Is there one of these that you would prefer?

**Capability**: “Sexual-well-being means being in control of one’s own sexual decision, sexual practices and sexual consequences including access to means of mitigating the risk related to sexual activities.”

**Subjective evaluation**: “Sexual well-being’ refers to the subjective, individual experience of sexuality and how someone experiences this in the context of his or her personal life and relational situation.”

**Capability+ subjective evaluation:** “Sexual well-being could probably be measured as self-perceived sexual health, which could include being comfortable or satisfied with one’s sexual identity and the motivation and ability to protect and enhance one’s sexual health (WHO)”

1. Assessing sexual well-being: You were asked what questions you would ask to understand how someone is doing in terms of their sexual well-being. Several important aspects were mentioned by you. I identified, for example, the ability to practice consent, ability to communicate about sex, ability to gain pleasure, ability access to sexual and reproductive health services (e.g. education, abortion, contraceptives), having knowledge/understanding etc. Or maybe you can identify other aspects when going through the answers. During the discussion, I will also provide an overview of measurements that are currently used in the literature and some critiques that come back in the literature.
   1. Are there aspects that you feel troubled about? Why?
   2. Are there certain aspects that you feel are more important than others? Why?
   3. Do you feel the relative importance of the aspects can differ between individuals? How?
   4. Do you feel the relative importance of the aspects can change for one individual? How?
   5. Can you imagine any life changes that might have influenced/ influence the relative importance of these factors?
   6. If you want to study sexual well-being/ improve sexual well-being, how can we deal with these different interpretations of sexual well-being or changes of the life-time?
2. Identifying determinants of sexual well-being: In this part we will draw an overview of the system behind sexual well-being.
   1. What are driving forces, what are restricting forces to improve sexual well-being in young people?
   2. How do these link to the different sub-construct of sexual well-being?
   3. What external factors are influencing what this system looks like?

### Part 2: Designing a framework.

Based on the discussions we had, we will think about how we can develop a framework to define and measure sexual well-being. What are the main takeaway messages we want to give to future researchers and policy-makers?

## Questions asked in round 3

### Feedback on the framework

1. When going through the report, I want to ask you to give some comments → You can just highlight them in the text
   1. Are there things that you do not agree with? For example, quotes that you would interpret differently, quotes that you feel are misplaced or statements I make that you disagree with…
   2. Are there quotes that you feel are inappropriate to use, for example are there quotes that are too sensitive, that you prefer not to have in the report.
   3. Are there things that you would want to rephrase?
2. After going through the report:
   1. Do you feel the proposed framework is complete? Are there aspects that missing or don’t get enough attention?
   2. Do you have any more comments on this framework?

### Feedback on the process

1. Process evaluation
2. What were your expectations from this research and were these fulfilled?
3. Were their aspects you particularly liked during the process. Are there aspects that could have been done better? (e.g. What did you think of the communication, the process report, the timing etc.)
4. The research was done in different rounds: An introduction session, a brainstorm session, a panel discussion and a feedback round. Do you feel there is an added value to having these separate rounds? Why/ why not?
5. Are there any other comments, thoughts, reflections you want to share?

# Annex 3: Responses Round 1

| Table 1: Results brainstorm | |
| --- | --- |
| **Defining sexual well-being** |  |
| Sexual well-being is about consent, feeling comfortable, accepted and safe | - Comfortable - Feeling comfortable in their own sexual life and health - Having positive views about one’s self sexually - Respect - Safety - Safe - Consent - Acceptance - Inclusion |
| Sexual well-being relates to pleasure and fun | - Being able to achieve satisfaction from one's sexual life - Experience of sexual pleasure - Pleasurable - Fulfilling - Enjoyable |
| Sexual well-being relates to freedoms and agency | - Empowerment - Freedom to express sexual orientation - Bodily autonomy - Free |
| Sexual well-being relates to knowledge and awareness | - Awareness - Understanding of sexual pleasure - Knowledge about safe sex practices with the skill to execute/negotiate about it - Good-quality information |
| Sexual well-being is a holistic concept and relates to mental, physical and social health | - Complete/whole, healthy, mental, social relations, body, emotional - Complete, state of health, reproductive system, functions - Healthy physically and internally (psychologically), or happy in connection to sexuality - Complete natural wellness, beyond physical look, emotional connection with our sexual orientation - A state of one's sexuality in relationship to his or her Social, physical and emotional ability. |
| Other | - Absence of disease |
| **Assessing sexual well-being** |  |
| Sexual well-being relates to consent, feeling comfortable, accepted and safe | - Are you feeling comfortable with your own sexual well-being? - What opinions do you have about yourself and body sexually? - Do you feel safe in your community to express your sexual identity - Do you understand and practice consent? - How do you express consent to sex? |
| Sexual well-being relates to communication | - Are you feeling comfortable talking about sex with someone? - Are there people with whom you can have discussion about your sexual experiences? - Are you feeling comfortable to talk about it with someone other than your partner/s, also with them? - How comfortable are you to discuss sexual well-being with your partner? - Do you feel that there are a significant number of individuals in your life that you can have open and honest discussions about your experiences with your sexuality or broader conceptualisations in society - Can you and how easily you communicate and negotiate safe sex with your partners? |
| Sexual well-being relates to pleasure and fun | - Are you experiencing sexual pleasure and satisfaction whenever you want it? - Are you satisfied by your sexual life? - Do you enjoy your sexual life? - Are you experiencing pleasure? |
| Sexual well-being relates to freedoms and agency | - Do you feel empowered to experience pleasure? - Are you able to enjoy pleasure and intimacy without pressure from society, influence of stereotypes, stigma, and discrimination - Are you able to freely choose your intimate partner? |
| Sexual well-being relates to knowledge, awareness, and safe sex practices | - Do you know the signs of any STI? - Do you know there is something called safe abortion? - Do you notice changes when it comes to your sexual health? - Do you have any idea of what sexual well-being is? - What can cause you to miss your period? - What is your level of understanding on sexuality education - Have you heard of “safe period” before? - Do you know how to track your menstrual cycle? - How often do you take STI tests? - Will you consider practicing safe sex? - How do you prevent pregnancy - What do you think of safe sex? Do you practice it? - Do you know what sexual act is and how does it conducted? |
| Sexual well-being relates to the state of sexual health in society e.g. accessibility of SRH services | - What are the indicators for abuse, STI rates? - Is abortion legal? - Do you have access to sexuality education? - Are you able to seek help? - Do you need help to find somebody to talk to? - Are you able to access the contraception of your choice? - Where do you find information? What information do you miss? How do you fill in your missing information? - How do you get informed on issues related to your sexual well-being? |
| Sexual well-being relates to attitudes and norms | - What are your views on service providers introducing contraceptives to young people? - Do you feel it’s appropriate to know anything about your sexual well-being? - Do you see sexual well-being to be of any importance? |
| Other | - How often do you engage in sexual activity? - Have you ever had a sexual relationship with anyone? - Have you at all touched, kissed or hugged the opposite sex? - Firstly, I will ask them: Have you scored less in exams before ? What did you do? I will definitely get someone to say “I cried or I was worried” then let them know that worrying and crying is part of your sexual well-being in relation to psychology. I will if anyone is talking to a friend here or how you feel when your teacher pairs you with the opposite sex. The answers will also let me know how well their physical relation is |
| **Sexual health versus sexual well-being** |  |
| Sexual health includes sexual well-being | - Sexual health involves sexual well-being. Sexual health is the complete state of sexual well-being - I think there’s a slight difference between the two, only because sexual health is the broad topic which has sexual well-being included - Sexual well-being is a subset of sexual health. |
| Sexual well-being includes sexual health | - I think they are interrelated, sexual well-being also encompasses sexual health - Sexual health focuses on only the health aspect, whereas sexual well-being focuses on the complete natural, physical, emotional, social well-being and health of an individual - Sexual health is a part of sexual well-being and influences sexual well-being |
| Sexual health and sexual well-being have a different focus | - Sexual health is more clinical, whereas sexual well-being takes on a more rights based approach. Sexual well-being has more life skills implications. - Sexual health for me is biologically and medically, and sexual well-being is more broad; it’s about expression, relationships, (body) confidence, mental health,etc. - Sexual well-being talks about the general state of sexual aspects, such as how health and pleasure are connected to create a good environment for someone to enjoy. Sexual health, is more related to the medical part of it, such as gyno controls, sti tests, protective measures. I do believe that they are related since the sexual health of someone can influence their sexual well-being and vice versa. - Sexual health focus on only the health aspect. Whereas sexual well-being focuses on the complete natural, physical, emotional, social well being and health of an individual - There is a difference, however, these two might be interconnected. Sexual health is purely related to biological state, while sexual well-being is a bigger concept, that also includes sexual health, relationships, bodily autonomy, freedom of expression, understanding pleasure, and etc. |
| Sexual and sexual well-being are the same/have the same goal | - I feel there is no difference between sexual well-being and sexual health. - I think sexual health and sexual well-being are related because they all seek to achieve the same goal thus; knowing yourself completely in relation to your sexuality. - To me sexual health and sexual well being are synonyms. They all mean the same state of one's physical, emotional and social-well being in relation to sexuality whereby not only the absence of disease but the ability to make a decision about your sexuality. |
| Other |  |
| **Intersectionality and sexual well-being** |  |
| ***Intersectionality influences the conceptualization of sexual well-being*** | |
| Intersectionality | - One's identity does influence one's sexual well being, be in regards to whom you are romantically and/or sexually attracted to; your kinks, your comfort level in certain sexual acts and how has sex and sexual well being perceived by you. Not only this what and how was sex introduced to you, its cultural implications or restrictions. - For me, I feel like my identify influence much because I define sexual well-being based on how I identify myself in all angles. It should be consensual, boy VS girl, in a private place and between adults. |
| Upbringing | - I'm coming from a conservative background, where sex before marriage was not considered good behavior. So this pressure was somehow following my personal and sexual development. - My personal identity influences how I interpret sexual well-being. In the sense that; growing up, my parents interpreted sexual well-being differently, hence changing my whole orientation about it. - I think everything can have an impact on our sexual well-being, for me, being raised in a conservative background, as well as an abusive one, my relationships(of any kind) are always based on safety, first of all, an important thing that I look for also in my sexual well-being. |
| Gender | - I'm female. We're often taught to believe that our sexual well-being is tied to a man's sexual well being and it takes a lot to unlearn that - Male - My sexual identity doesn't influence how interpret my sexual well-being. Not all. - Female- Yes it does influence my interpretation of sexual well-being |
| Sexual identity | - Being queer changed my perception of sexual well-being and what pleasure can look like. |
| ***Intersectionality impacts sexual well-being*** | |
| Intersectionality | - All the aspects stated above define my personal identity and I feel like they influence my sexual well-being. I think that every aspect in someone’s life influences their well-being and therefore also their sexual well-being. I base myself for this on intersectionality. |
| Gender | - My gender and sexual identify as a heterosexual woman influence my sexual well-being because of the risk this puts me in terms of sexual and domestic violence |
| Personality | - Open , welcoming , secure. It allows to be able to have good view about myself sexually since I see the world to be different in everyone’s. It allows me live my truth about myself since I believe sexual well being varies from person to person - Self-confidence is my definition of personal identity. My identity influences all I do. Including my sexual well-being. - Committed, cooperative, devout, disciplined, open-minded , assertive. I feel it will definitely have an influence because I can't identify as one thing and be advocating for another which in turn will contradict my beliefs and values or what I identify as. |
| Health state | - Health conditions also play a big role in sexual well-being, for example hormones can change a lot, being aware of the state of your sex hormones is quite important |
| **Defining sexual activity** |  |
| Sexual activity encompasses different elements | - Consent - Intimacy - Pleasure - Satisfaction - Bonding - Arousal |
| Sexual activity is not only limited to penetrative sex or activities that lead to an orgasm | - Kissing, fondling, caressing, penetration, oral sex, or contact with any other parts of the body to cause sexual excitement - Activities which result in pleasure, but not necessarily resulting in orgasm. - Sexual activity is when people engage in acts for the purpose of sexual pleasure. - Sexual contact that results in arousal and that is consensual - Any activity meant to provide pleasure or orgasm to an individual not only through penile-vaginal sex but self pleasure (mutual masturbation), phone sex, frottage, oral and anal sex. - My definition would be based on general sexual orientation, sexual health, and its related activities. But not limiting it to only sexual activities as in “sexual intercourse” |
| Sexual activity is not necessarily limited to romantic relations | - Maybe done alone or with one or more partners - Very important to not associate sexual activity with romance, love and intimacy |
| Sexual activity can have different meanings for different people | - Not limited only to penetration and can be defined freely by every person - People's own conceptions of what sex is- for example a recognition that what people define as sex- some define it as penis in vagina intercourse some as any sexual contact |
| Other | - I will prefer to use reproductive health instead of sexual well-being to get people to buy in to what I want to share with them - Canal knowledge |

# Annex 4: Narrative sexual well-being framework

## Sexual well-being ecosystem

This framework urges refocusing the conceptualization of sexual well-being from a normative individual approach to a sexual well-being ecosystem approach. At its core, the sexual well-being ecosystem is holistic and intersectional.


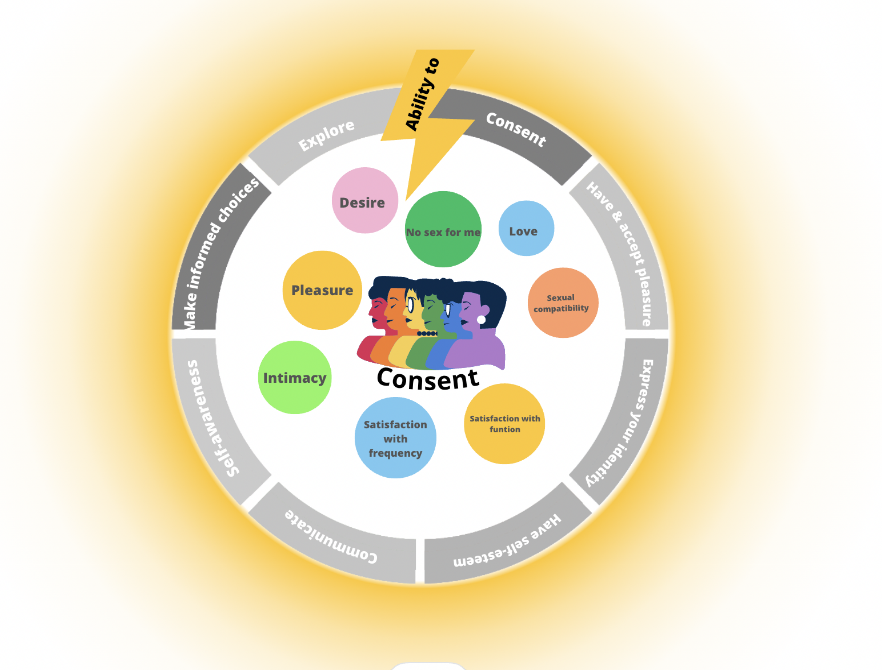
This means it acknowledges that, at an individual level, sexual well-being can mean different things for different people. For some individuals, it might mean finding intimacy in relations, while for others, sexual well-being can mean having no sex at all. Different elements can be of different relative importance at different moments in someone’s life. We emphasize the importance of not rigidly defining sexual well-being at the individual level. It is also essential to leave room for diverse interpretations of what “intimacy” and “sexual activity” means, and move away from the normative idea that sex can only mean penetrative sex, and intimacy can only be physical. This enables, e.g. queer people and people living with certain disabilities, to redefine what sexual activity is, without being assessed as abnormal. Consent is the only element that needs to be predefined.

It also means that sexual well-being should not be understood exclusively from the subjective perspective of the individual. Rather, sexual well-being should be thought of as the ability of an individual to consent, to explore, to be self-aware about their sexual health and what makes them feel comfortable. They need to be able to have and accept pleasure, to communicate what they find important, to be comfortable before, during, and after sexual activity, but also being comfortable with who they are and have self-esteem. They need to be able to express their sexual identity. Therefore, a complex system around the individual, needs to be taken into account. For an individual to be able to grow, understand and pursue their desires and preferences, enabling environments need to be created. Therefore, sexual well-being also means:


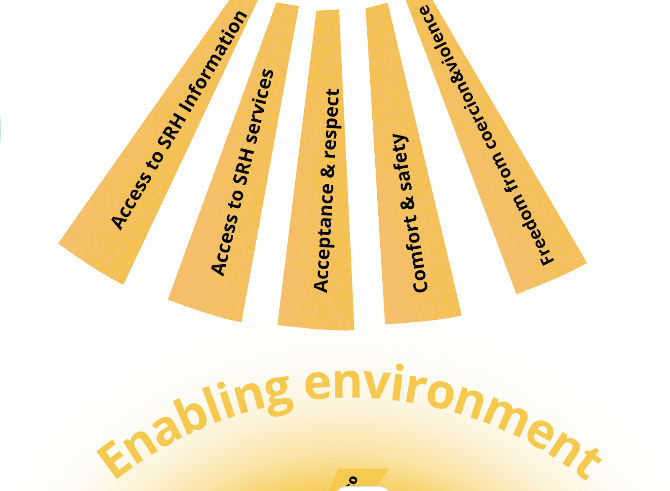


*Having access to SRH information and services.* Information and services need to be accessible to understand and manage your sexual health. A wide variety of information should be provided, both through accessible modern media and sexuality education. Information encompasses knowing how to have safe sex, being trauma-informed, having knowledge about your body, your boundaries, how to access SRH services, and how to improve your sexual health if you would want to. Understanding how harmful norms, negative experiences such as trauma, bullying, and sexual violence, and your mental and physical state might affect your sexual health and how to manage this, are essential to enable sexual well-being. This also includes having knowledge on the wide variety of relationship forms and sexual activities, and how different individuals might need different things to feel comfortable to gain pleasure. Appropriate sexual health facilities, contraception, and abortion services can limit the mental burden of sexual problems, STIs and unwanted pregnancies, and enable individuals to be more comfortable before, during, and after sexual activity.

*Being accepted & respected.* For a person to be comfortable in their sex lives, to be able to explore and to truly consent, they need to be accepted and respected for who they are, what they look like, and what their sexual preferences are. This also includes being accepted and respected in the online world.

*Having comfort & safety .* People should feel comfortable and safe in society to communicate about sex in the community, to express their sexual orientation, to accept themselves as a sexual being, and to pursue their needs and preferences. This safety also includes safety in the online world. Comfort and safety also relates to the sexual and reproductive rights someone has.

*Freedom from coercion and violence.*To be able to consent, to make informed choices and pursue the sex life they want, people should be free from coercion. More specifically, this includes but is not limited to freedom from physical coercion, freedom from harmful societal norms, and freedom from grooming. Violence, which includes sexual violence, cyber violence and bullying, can limit a person’s ability to have and accept pleasure, to explore, to consent, to have self-esteem and be comfortable.

All of these factors are of course interrelated, and connected.

## Dark clouds in the sexual well-being ecosystem


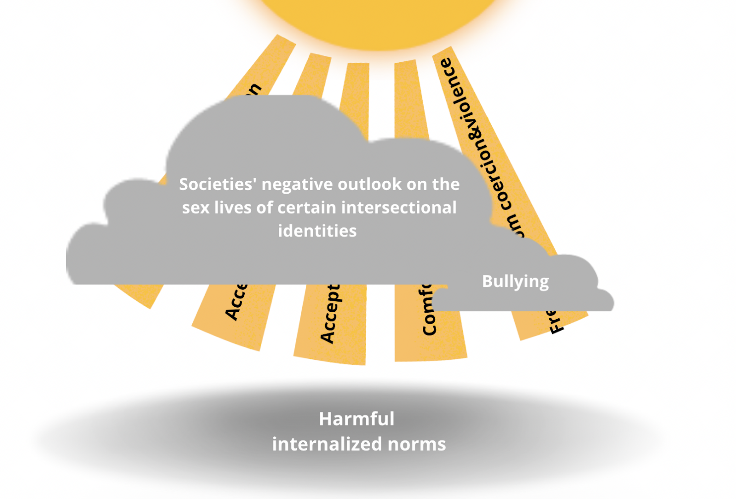
There are dark clouds in the sexual well-being ecosystem that make sexual well-being not equally accessible for everyone. First of all, societies' negative outlook on the sex lives of individuals, limits the accessibility of their sexual well-being. While some societies are really permissive and create safe and comforting environments, other societies limit the rights of different groups, making it unsafe and not comfortable for different individuals to have sex lives. Also bullying, which includes cyberbullying, can limit certain individuals to access sexual well-being. Harmful norms and violent messages, that are spread through porn, peers, media, and legal prosecution systems, are internalized, and limit the individual’s ability to consent, have and accept pleasure, explore, be comfortable, and have self-esteem. Harmful norms include messages such as, sex is supposed to be painful, sex only includes penetrative sex, sex and pleasure are shameful and wrong, sex is an obligatory part of a good relationship, sex is not something you are supposed to talk about.

Again, it is important to acknowledge that certain groups are more affected by this than others. Racist, heteronormative and ableist societies have less availability of suitable information and services for ethnic minorities, LGBTQIA+, and people living with disabilities. In societies where sex workers and LGBTQIA+ are illegal or stigmatized, they are not accepted and respected and their safety and comfort is compromised. The responsibilities of pregnancy prevention, and the high consequences of unwanted pregnancies, mainly affect women’s comfort and safety. Young people, women, queer people, people with disabilities, sex workers are more often victims of harmful norms in society. Often these norms are more restrictive towards these groups having sex lives, compared to others.

## Sexual well-being is the state’s responsibility


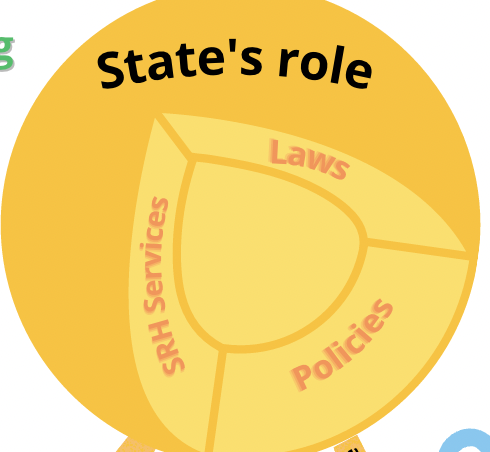
Shifting the framing of sexual well-being from an individual to a system’s conceptualization, allows emphasizing the responsibility of the state. The state has to play an active role in creating enabling environments. First of all, through the provision of laws, policies and infrastructure. Some examples are anti-discriminatory laws, abortion laws, youth-inclusive policies. Secondly, by assessing and addressing society’s negative outlook towards the sex lives of certain groups, for example women, young people, people with disabilities, sex workers. These two roles go hand-in-hand and create equal opportunities, for every individual, to understand, define and improve their sexual well-being.


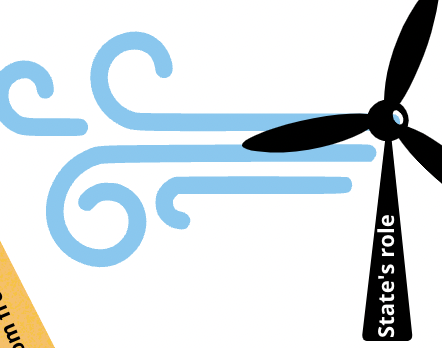

Supplement: Annexes 1-4 [file ZRHM_A_2474337_SM4136.docx]
